# Development of a Whole Slide Imaging System on Smartphones and Evaluation With Frozen Section Samples

**DOI:** 10.2196/mhealth.8242

**Published:** 2017-09-15

**Authors:** Hong Yu, Feng Gao, Liren Jiang, Shuoxin Ma

**Affiliations:** ^1^ Department of Pathology Center Shanghai General Hospital Shanghai Jiao Tong University School of Medicine (originally named “Shanghai First People’s Hospital”) Shanghai China; ^2^ TerryDr Info Technology Co., Ltd Nanjing, Jiangsu China

**Keywords:** mobile health, image processing, cloud computing for health care, whole slide imaging

## Abstract

**Background:**

The aim was to develop scalable Whole Slide Imaging (sWSI), a WSI system based on mainstream smartphones coupled with regular optical microscopes. This ultra-low-cost solution should offer diagnostic-ready imaging quality on par with standalone scanners, supporting both oil and dry objective lenses of different magnifications, and reasonably high throughput. These performance metrics should be evaluated by expert pathologists and match those of high-end scanners.

**Objective:**

The aim was to develop scalable Whole Slide Imaging (sWSI), a whole slide imaging system based on smartphones coupled with optical microscopes. This ultra-low-cost solution should offer diagnostic-ready imaging quality on par with standalone scanners, supporting both oil and dry object lens of different magnification. All performance metrics should be evaluated by expert pathologists and match those of high-end scanners.

**Methods:**

In the sWSI design, the digitization process is split asynchronously between light-weight clients on smartphones and powerful cloud servers. The client apps automatically capture FoVs at up to 12-megapixel resolution and process them in real-time to track the operation of users, then give instant feedback of guidance. The servers first restitch each pair of FoVs, then automatically correct the unknown nonlinear distortion introduced by the lens of the smartphone on the fly, based on pair-wise stitching, before finally combining all FoVs into one gigapixel VS for each scan. These VSs can be viewed using Internet browsers anywhere. In the evaluation experiment, 100 frozen section slides from patients randomly selected among in-patients of the participating hospital were scanned by both a high-end Leica scanner and sWSI. All VSs were examined by senior pathologists whose diagnoses were compared against those made using optical microscopy as ground truth to evaluate the image quality.

**Results:**

The sWSI system is developed for both Android and iPhone smartphones and is currently being offered to the public. The image quality is reliable and throughput is approximately 1 FoV per second, yielding a 15-by-15 mm slide under 20X object lens in approximately 30-35 minutes, with little training required for the operator. The expected cost for setup is approximately US $100 and scanning each slide costs between US $1 and $10, making sWSI highly cost-effective for infrequent or low-throughput usage. In the clinical evaluation of sample-wise diagnostic reliability, average accuracy scores achieved by sWSI-scan-based diagnoses were as follows: 0.78 for breast, 0.88 for uterine corpus, 0.68 for thyroid, and 0.50 for lung samples. The respective low-sensitivity rates were 0.05, 0.05, 0.13, and 0.25 while the respective low-specificity rates were 0.18, 0.08, 0.20, and 0.25. The participating pathologists agreed that the overall quality of sWSI was generally on par with that produced by high-end scanners, and did not affect diagnosis in most cases. Pathologists confirmed that sWSI is reliable enough for standard diagnoses of most tissue categories, while it can be used for quick screening of difficult cases.

**Conclusions:**

As an ultra-low-cost alternative to whole slide scanners, diagnosis-ready VS quality and robustness for commercial usage is achieved in the sWSI solution. Operated on main-stream smartphones installed on normal optical microscopes, sWSI readily offers affordable and reliable WSI to resource-limited or infrequent clinical users.

## Introduction

Virtual slides (VSs) generated from whole slide imaging (WSI) systems are an essential component of digitized diagnostic processes, as they provide extended fields-of-view (FoVs) under microscopes without handling specimens physically [1,2]. In addition to providing a basis for automated analysis [3-5], the reliability and efficiency of VSs is widely considered on par with traditional light microscopy (or even superior in certain cases [6-9]), making it one of the main trends in both digital pathology and bioinformatics [10-12]. The conversion from direct observation to digitization has been dramatic [13], even though the technology is still under heavy development and shows performance bottlenecks that may not be broken in the near future [14,15].

In practice, however, automated scanners that are commonly used to capture and process such data cost approximately US $50,000 or more up-front, even for low-frequency usage. In many developing countries, this financial obstacle alone has significantly impeded modernizing related departments in hospitals [16], such as that of pathology in China. Lacking digitization undermines productivity and diagnostic accuracy, commonly leading to poorer administrative attention and tighter budgets, thus forming a vicious cycle.

In recent years, two alternative solutions have attracted much academic and commercial interest. One solution is aborting the automation feature, thus leaving the operator to control the microscope manually, reducing the product package to a dedicated digital camera and software [17-19], and costing as little as US $10,000. Other attempts have made use of smartphones, which not only have integrated capturing and processing abilities, but are also widely distributed among clinical professionals, thus lowering the start-up cost to near zero. A small number of products in the latter category in both research and commercial stages have been evaluated by clinical professionals [20], but to the knowledge of the authors, all are made exclusively for relatively expensive iPhones and are not yet commercially available. Although rarely explained explicitly, robustness of full automation and guarantees of successful VSgeneration could be serious obstacles between publishable research and commercial products. Additionally, diversity in hardware and operating systems might be the reason that Android phones, although dominating handset markets in developing countries, are largely ignored.

In this paper, a WSI system on mainstream smartphones that just became publicly available with commercial-quality and low cost (named scalable WSI; sWSI [21]), is introduced and evaluated. sWSI offers fast and reliable WSI on most handsets, average Androids or flagship iPhones alike, reducing the up-front cost to approximately US $100 and the average service cost per scan is as low as US $1. Pathologists recognize sWSI as an attractive alternative to stand-alone scanners, especially for quick scans (eg, frozen sections) as well as medium/low-frequency usages.

Beyond technical development of the sWSI system, this research also included evaluating it with cryosections, also known as the frozen section procedure. Cryosectioning is widely used in oncological surgeries, which require significantly faster preparation and diagnoses compared to traditional histology techniques. Frozen section samples have much lower technical quality, making them very difficult to analyze for whole slide scanners [22], but this process offers a good challenge to test the limits of sWSI.

## Methods

### System Overview

**Figure 1:**
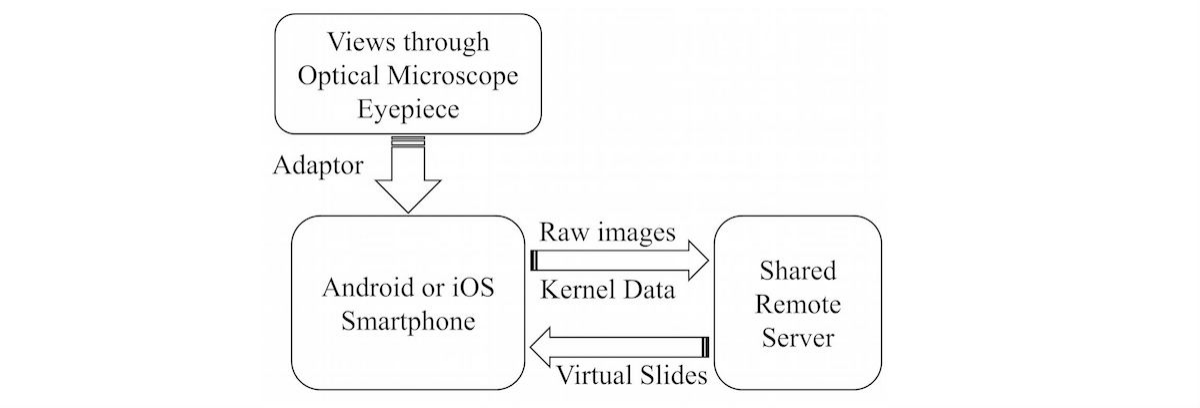
Simplified sWSI solution structure.

There are two essential and costly components in a typical WSI scanner: the capturing unit, typically a set of lenses with a distortion-calibrated digital eyepiece; and on-board or external high-performance computers. Like any dedicated devices, since both parts are specifically built for the system, they are mostly nonproductive when the system is idle and thus waste much of their value when underused. Unfortunately, this is commonly the case for smaller hospitals in which complicated pathological diagnoses occur only occasionally. This situation, coupled with consumer electronics' performance approaching that of medical-grade tools, led to the idea of creating sWSI with the structure illustrated in Figure 1.

#### Hardware

To provide full WSI functionality at a dramatically lower cost, sWSI aims to reduce the cost of both hardware necessities. For the optical part, sWSI reversibly upgrades existing microscopes with built-in cameras of smartphones and compensates for the unknown optical distortions computationally, as discussed in detail in the *On-the-fly Image Distortion Correction* subsection below. For the computing part, sWSI utilizes smartphones for light-weight real-time processing and transfers the major bulk of the work to shared remote servers to allow temporal-multiplexing for improving utilization rate and cost-sharing.

**Table 1:** Minimal and optional hardware specifications.

Item	Minimal Specification	Recommended Specification
Operating System Version	Android 4.2 or iOS 9	N/A
Central Processing Unit	Dual-core @ 1.2 GHz	Quad-core @ 2.4 GHz
Camera	3 megapixels	12 megapixels

Although the prices of mainstream smartphones vary widely, much of the cost comes in the form of user-friendly features (eg, security or battery life) that are largely irrelevant to sWSI. Thanks to the fast expansion of smartphone markets, their cameras, which used to be the critical link in such clinical applications, are now on par with main-stream dedicated digital eyepieces [23,24]. Overall, newer smartphone models can easily meet the minimal requirements listed in Table 1 at prices as low as US $100. It should also be noted that the higher-end smartphones that meet the optional specification for better performance may be bought at deep discounts as *used* or *refurbished*, which may suffer short battery life or a repaired screen, but do not affect the performance of sWSI.

**Figure 2:**
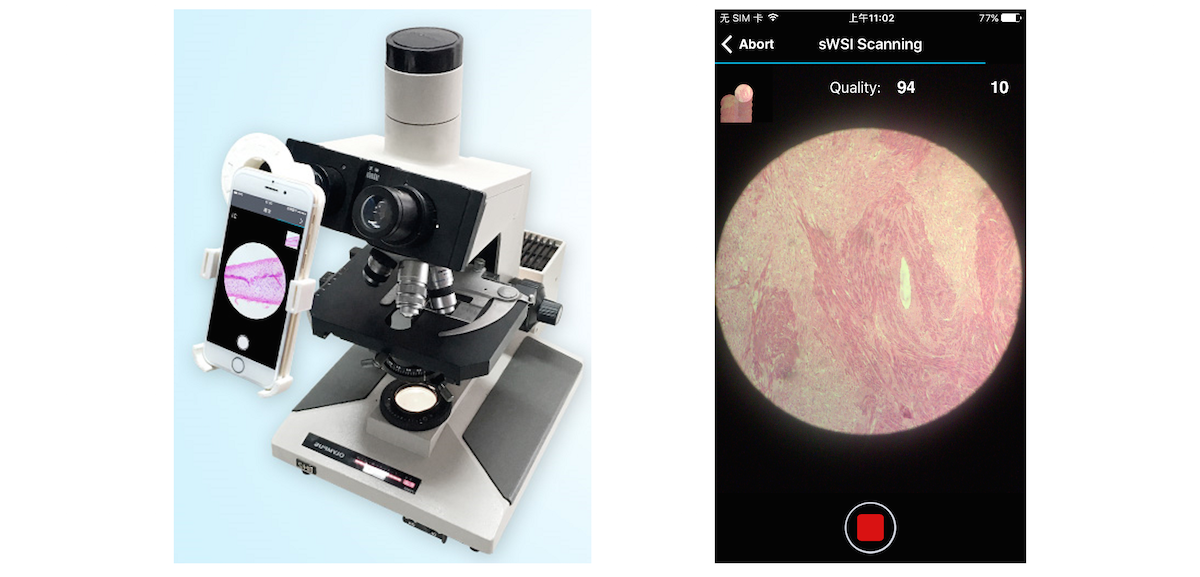
Typical hardware setup (left) and user interface (right).

Most professionals in research and health care services already own a handset that meets the criteria listed in Table 1, so sWSI requires installing only one adapter for each pair of existing smartphone and optical microscope. These microscope-smartphone adaptors are available with many commercial options as well as open-source designs for do-it-yourself 3-dimensional printing [25], although the ones specifically built for each phone model are preferred, to minimize the need for adjusting camera-eyepiece alignment and to block disrupting light sources. One setup is demonstrated in Figure 2, utilizing a used iPhone 6 costing US $200 installed on an Olympus BH2-BHS microscope with a scalScope adapter, which took about 15 seconds to set up, and was used for the clinical evaluation discussed later.

#### Software

In addition to image compressing, transferring and VS synthesizing is needed in any whole slide scanning systems, and the software in sWSI is also responsible for automatically measuring and compensating hardware diversity. Unfortunately, fully localizing many of these functions is beyond the reach of mass produced mobile devices. Synthesizing the VS from FoVs requires at least several gigabytes of random access memory and sequentially processing hundreds of FoVs at full resolution can take an hour or more on a mobile central processing unit (CPU). The VSs will be stored remotely anyway, so there is little extra cost in moving the bulk of processing onto remote servers, as implemented in sWSI. The downside of this distributed computing model is introducing significant risks of failure by splitting the processing work-flow into asynchronous halves, but in sWSI this is solved, as explained in *Fail-Proof Distributed Processing* subsection below.

Another practical issue worth noting is that due to architecture and driver support issues beyond the scope of this paper, most Android phones only support JPEG image capture at higher resolution, which cannot be directly processed pixel-by-pixel. This issue significantly constrains data flux since each FoV taken must go through an extra encoding-decoding routine costing several hundred milliseconds, depending on CPU power and resolution. As a result, the sWSI Android app limits the capturing resolution to approximately 3 megapixels and generally achieves throughput of approximately 1 to 3 FoVs per second, except for rare models with drivers offering high-resolution pixel data of images captured.

### Fail-Proof Distributed Processing

#### Basic Scan Procedures and Interaction

**Figure 3:**
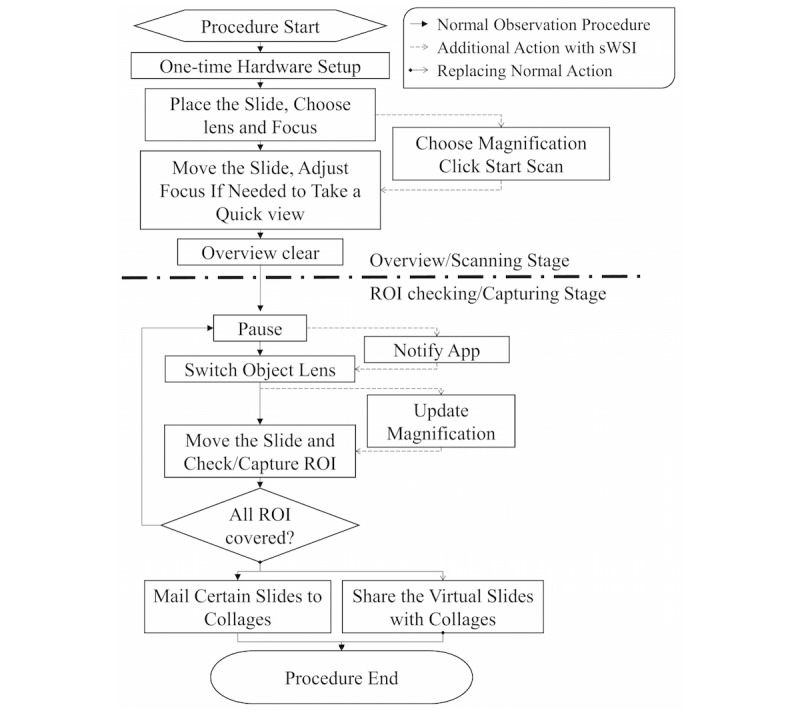
Microscope observation procedures adapted for sWSI. ROI: region of interest.

In sWSI, a smartphone client app is responsible for gathering user inputs, capturing and processing the FoVs, and guiding users interactively, with a user interface during the scan (Figure 2). There is very little difference between the scanning procedure with sWSI shown in Figure 3 and that practiced by most microscope users, except for choosing a few parameters.

**Table:** 

Error code	Description
Moving too fast	The translation is too far, so the KP matching in SURF may be unreliable
Lost	No reliable translation can be obtained: the causes cannot be further distinguished by the machine but should be noticeable to the users, such as moving so fast that there is little overlapping between the current pair of FOVs or the camera is out of focus
Touching a boundary	There are few KPs detected, so the FoV is likely near a boundary
No error	The translation is reliable

#### Real-Time Feedback on Clients

The client's share of processing focuses on speed and robustness instead of accuracy, and therefore uses down-sampled copies of camera input. An algorithm roughly estimates pairwise translation of FoVs by stitching each captured FoV with the last one through key point (KP) detection and matching with the speeded-up robust features (SURF) algorithm [26,27]. This translation is then used in three ways: updating a mini-map illustrating current location on the slide, feeding a finite-state machine to manage the kernel asynchronously, and providing feedback to users as guidance for operating the microscope. The feedback and their trigger descriptions are listed in Table 2.

With users following the hints, sWSI essentially creates a closed feedback loop that allows scan-time interference against potential failure, such as inability to focus properly on thick samples or to track positioning on barren regions. This mechanism prevents most flops due to sample preparation and user operation before spending a long time in completing the scan, which is a common issue with automatic scanners.

#### Full Resolution Processing With A-Priori Knowledge on Servers

The cloud servers in sWSI are the primary powerhouses of computation. With full resolution FoVs and scan results from clients, servers restitch the adjacent FoVs at maximal accuracy, correct distortion, and generate the virtual whole slide. The asynchronous two-staged stitching performed respectively on the clients and servers, however, has inherent weak spots on both stability and efficiency.

The FoV pairwise stitching is based on KP detection and matching, whose outcome in turn is resolution-dependent. As a result, such outcomes in down-sampled and original resolution may potentially be significantly inconsistent. In many cases, as in almost every VS constructed from 100 FoVs or more with prototypes of sWSI, the full-resolution stitching produced unreliable matching at least once.

Conversely, by the law of large numbers, it is desirable to match as many KP pairs as possible for accurate estimation of the FoV-wise matching function, especially where this function has high degrees of freedom (as is the case of sWSI where raw images are nonlinearly distorted in unknown patterns). The computational cost of brute-and-force KP matching, however, grows quadratically with the number of KPs.

**Figure 4:**
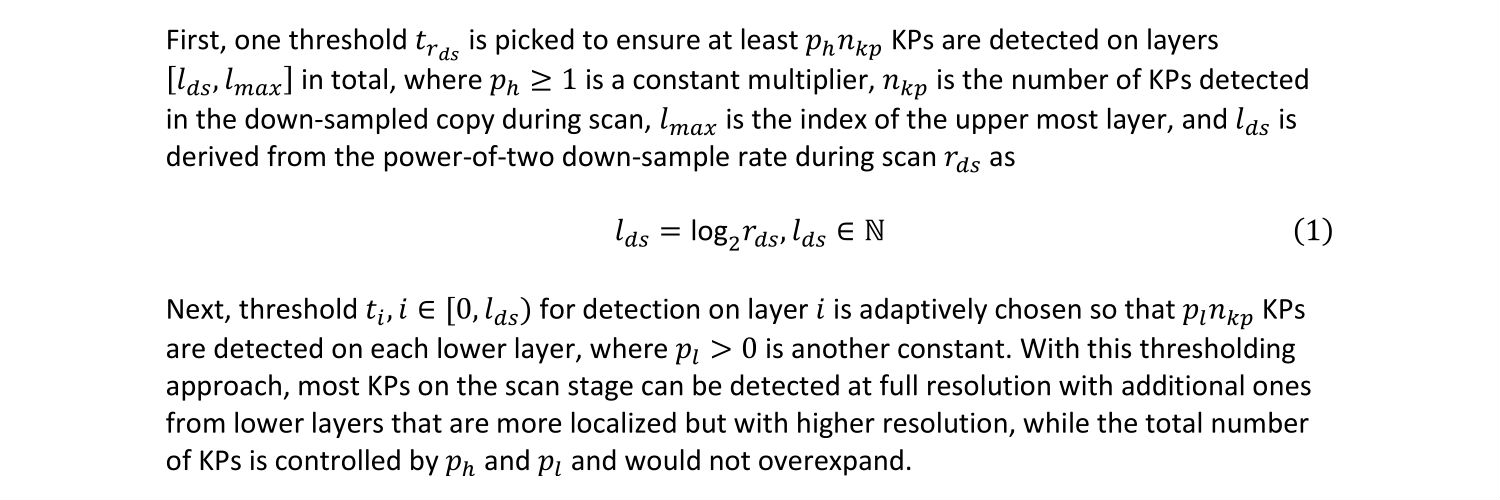
Adaptive thresholding in SURF.

To resolve both issues at once, sWSI employs an a-priori knowledge-based SURF KP detection and a matching algorithm on the server. SURF detects KPs from a virtual image pyramid that has lower resolution on higher layers. In sWSI, instead of detecting with one threshold across all layers, multiple thresholds are chosen adaptively as described in Figure 4.

**Figure 5:**
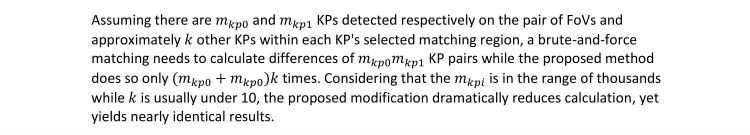
Computational benefits of selective KP matching.

Afterwards, instead of brute-and-force matching by calculating difference of all pairs of KPs and picking the optimal set of matches, sWSI selectively calculates those within a constant distance from the coordinates indicated by the scan stage translation with up-sampling and assumes all others are infinitely large. Figure 5 further details this process.

Since KP matching takes a large number of float point operations and consumes a large portion of time, this reduction boosts the overall efficiency of sWSI by over 50%.

#### On-the-Fly Image Distortion Correction

When stitching each FoV pair to match KPs, the projection function can be in any format as long as it minimizes error without overfitting. Combining all FoVs into a single continuous view, however, requires the projection to be linear so the nonlinear distortion must be corrected first. If the distortion is not corrected, the order of the stacked-up nonlinear transfer function of each FoV onto the whole slide will keep growing by each FoV and become very inefficient to solve.

**Figure 6:**
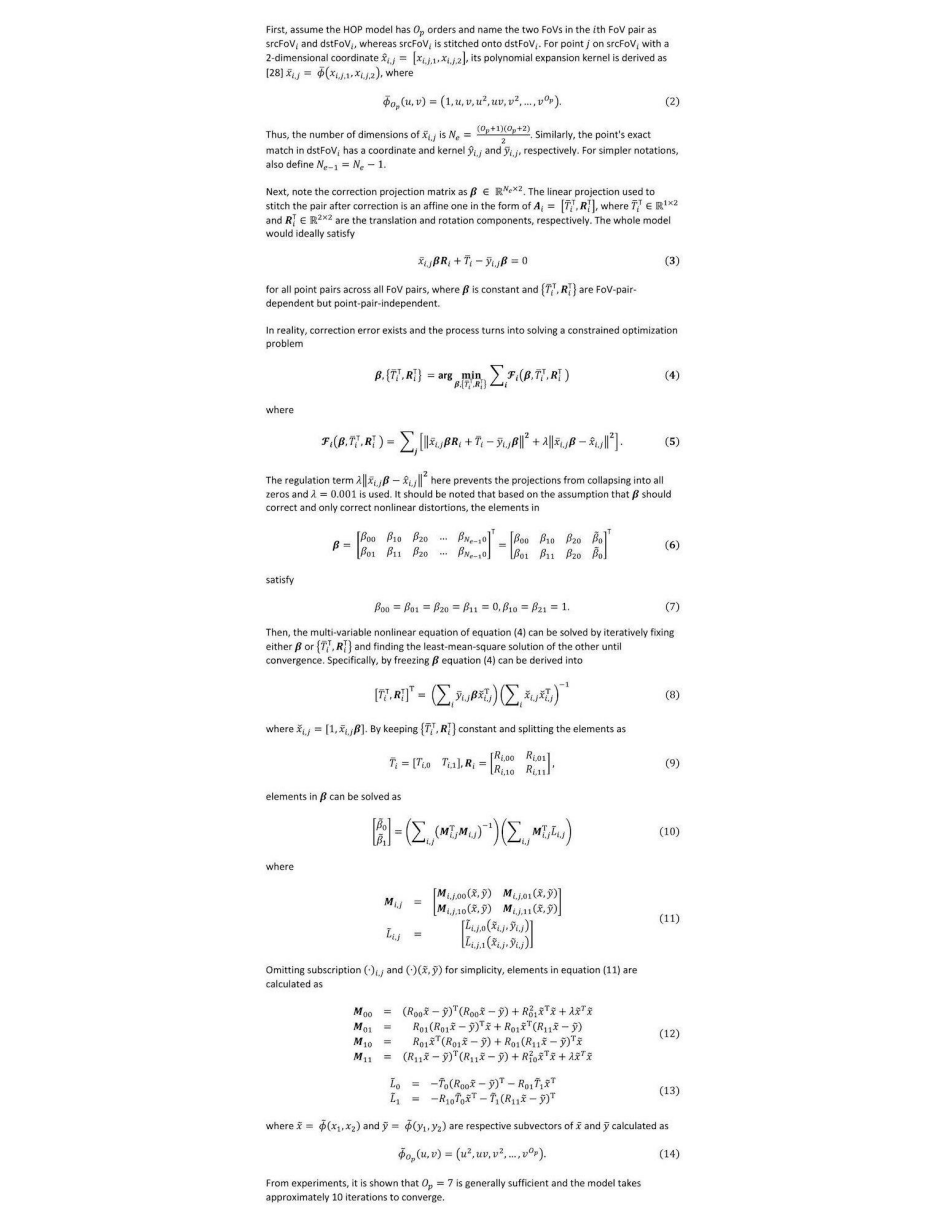
On-the-fly distortion correction based on matched KPs.

Designed to fit any combination of microscope and smartphone model, sWSI assumes a generalized high-order polynomial (HOP) inverse-distortion model [28], which mathematically approximates any function with marginal error if the order is sufficiently high, as proven by Taylor's theorem [29]. Specifically, it is assumed that there exists a constant but unknown HOP projection function for each scan procedure that maps the raw FoVs into a corrected 2-dimensional space, where any matched pixel pairs in overlapping FoVs share the same phase difference for that FoV pair. After the raw FoVs are corrected by a HOP function, each adjacent FoV pair can be stitched with translation onto each other with small error. In sWSI, this HOP projection matrix is solved iteratively based on FoV-pair-wise KP matching, formulated as described in Figure 6.

### Clinical Evaluation Setup

To assess the diagnosis-readiness of sWSI VSs of challenging cases, a clinical evaluation experiment was carried out in Pathology Center, Shanghai General Hospital, School of Medicine, Shanghai Jiaotong University (SJTU-PC) between February 23rd, 2017 and April 15th, 2017. A total of 100 frozen section slides (from one of the five categories listed in Table 3) gathered among inpatients between February 23^rd^, 2017 and April 1st, 2017 were randomly selected as the test dataset. The slides varied significantly in size and shape, were prepared routinely by technicians in the department, and may have had common issues with frozen sections, such as unevenness and folding. Consideration of both possibilities of tumors as well as benign lesions were included in the diagnosis, following generally accepted practice standards [30-32].

**Figure 7:**
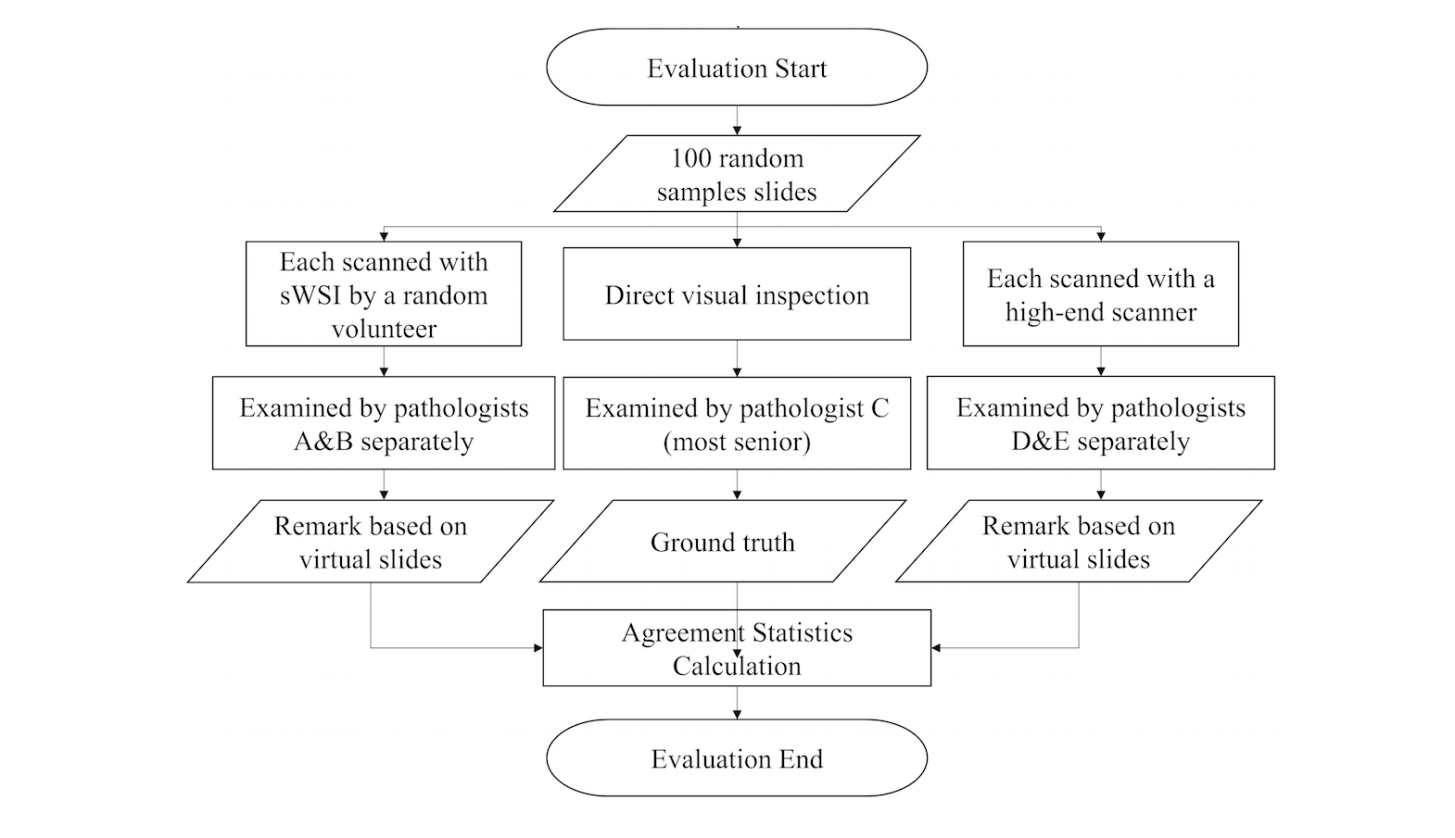
sWSI clinical evaluation procedure.

All samples went through three diagnosis procedures using optical microscopy, sWSI VSs, and high-end standalone scanner VSs under a 20X objective lens. In each procedure, each sample was examined by one or two pathologists independently. These diagnoses were then compared against each other to obtain agreement statistics. The full procedure is illustrated in Figure 7. This would be the first of a planned sequence of experiments evaluating sWSI on a wide range of samples, so we focused on using iPhones (on which the international version of sWSI is supported). The smartphones used were: one iPhone 5s, one iPhone 6, and one iPhone 6 plus, purchased from the second-hand market between US $120 and $200. The microscopes for sWSI scanning were: an Olympus BH2-BHS, an Olympus CX21, and a Phoenix PH50-1B43L-PL, all being low-end bio-microscopes. The scanner used in this experiment was Aperio AT2 from Leica, which is a high-end model. The microscope for optical microscopy was an Olympus BX51, which is another high-end model. Pathologists A, B, C and D are all senior faculty members from SJTU-PC, while the work of pathologist E was carried out by 10 senior and respected pathologists invited from top hospitals across China, as listed in *Acknowledgements*.

**Table 3:** Sample categories, counts, and notes.

Category	Counts	Notes
Breast	20	Requiring median image quality
Uterine corpus	20	Requiring median image quality
Thyroid	28	Requiring high image quality; one sample of scanner VS missing
Lung	31	Requiring very high quality; one sample of sWSI VS missing (physically damaged)
Ovary	1	Accidentally included, as it was assumed to be a thyroid sample; diagnosis only considered in calculating overall statistics

As part of the evaluation, quantifying the learning curve of manual scanning with sWSI was covered in this experiment. The VS quality depends on the sample being in focus and even the scan speed in the scanning process, so it is positively linked to the level of skill of operator or inversely to the time consumed. Presumably, as the operator gains experience, they would scan faster while maintaining the same quality. An intuitive way to measure this assumption is by comparing the time consumption of carefully scanning a unit area of samples under a fixed objective lens magnification against the number of slides scanned by operator. To this end, a total of 15 interns, medical school students, and assistant technicians who were well trained to use microscopes but had no prior experience with sWSI volunteered to scan the slides with sWSI. With duplication, each participant scanned 10 of the 100 slides after watching a demonstration, with the approximate sample size and time consumption recorded. Only one of the duplicated scans of the same sample was randomly selected for accuracy evaluation.

## Results

### Sensitivity and Specificity

The accuracy of diagnosis in this evaluation experiment was measured on a slide-wise level, since the classifications of regions in the sample can be subjective and pathologists usually consolidate multiple patterns across the whole sample into a conclusion. The sensitivity is defined as whether a pathologist can use a VS to correctly identify all critical regions of interest across the sample. The specificity is defined as whether the pathologist can correctly analyze each region of interest and identify patterns based on a VS.

To represent the performance based on the sensitivity and specificity defined above, each diagnosis was classified into one of three categories: accurate, low sensitivity (LSen), or low specificity (LSpe). For each VS, if the pathologist missed any critical region of interest, the VS was deemed as LSen. With the VS past the sensitivity check, if the pathologist then correctly identified patterns of all regions of interest, it was classified as being accurate; otherwise it was LSpe. When averaged, these metrics were weighted by their sample counts.

**Figure 8:**
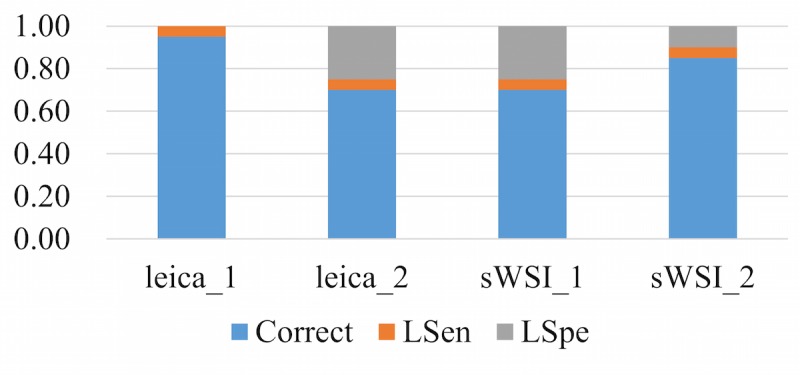
Performance based on scanner and sWSI virtual slides. Sample type: breast. LSen: low sensitivity; LSpe: low specificity.

**Figure 9:**
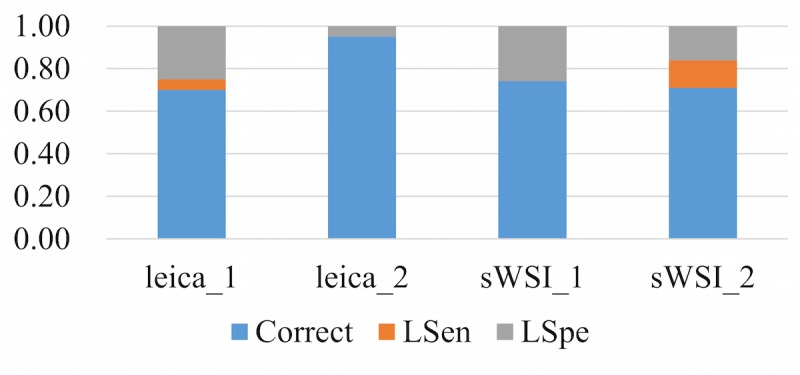
Performance based on scanner and sWSI virtual slides. Sample type: uterine corpus. LSen: low sensitivity; LSpe: low specificity.

**Figure 10:**
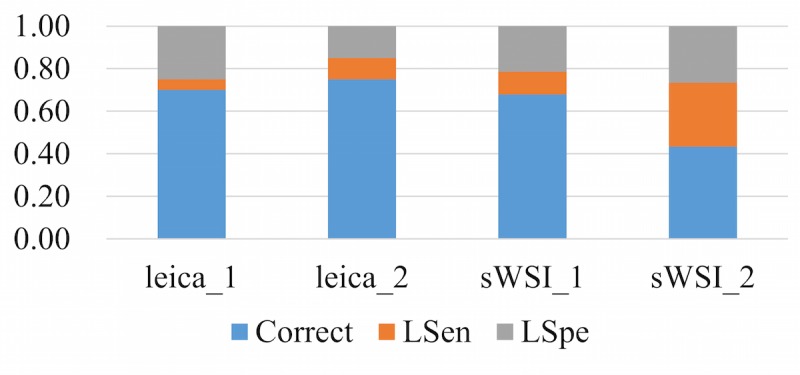
Performance based on scanner and sWSI virtual slides. Sample type: thyroid. LSen: low sensitivity; LSpe: low specificity.

**Figure 11:**
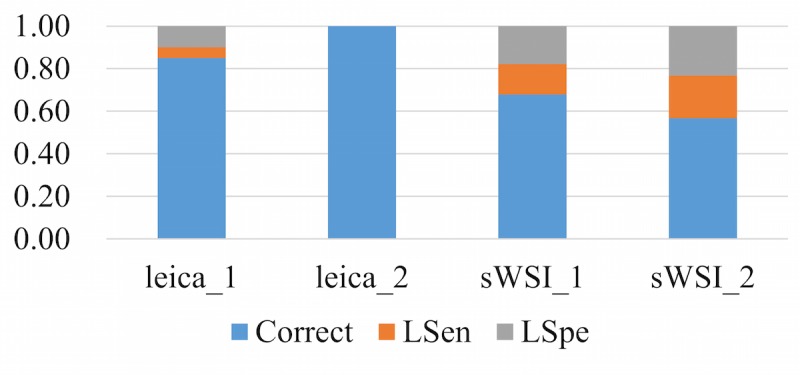
Performance based on scanner and sWSI virtual slides. Sample type: lung. LSen: low sensitivity; LSpe: low specificity.

**Figure 12:**
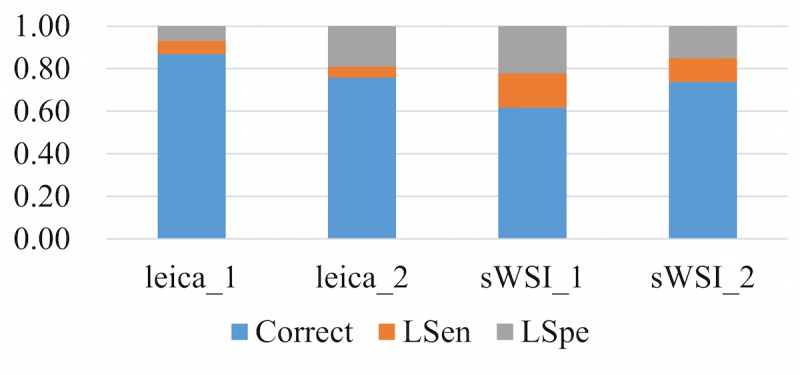
Performance based on scanner and sWSI virtual slides, averaged. LSen: low sensitivity; LSpe: low specificity.

**Figure 13:**
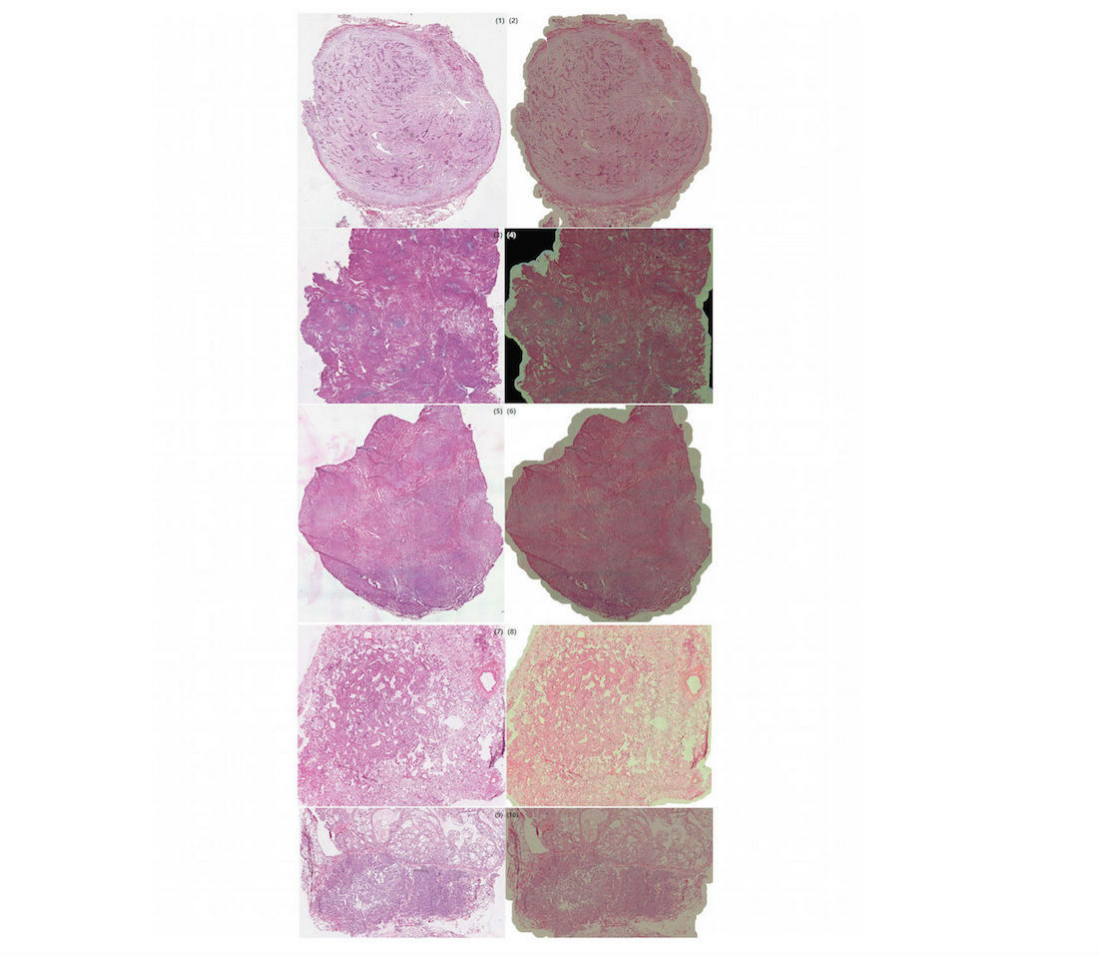
Selected virtual slide regions from sWSI (right) with good quality, compared to those from the Leica scanner (left).

**Figure 14:**
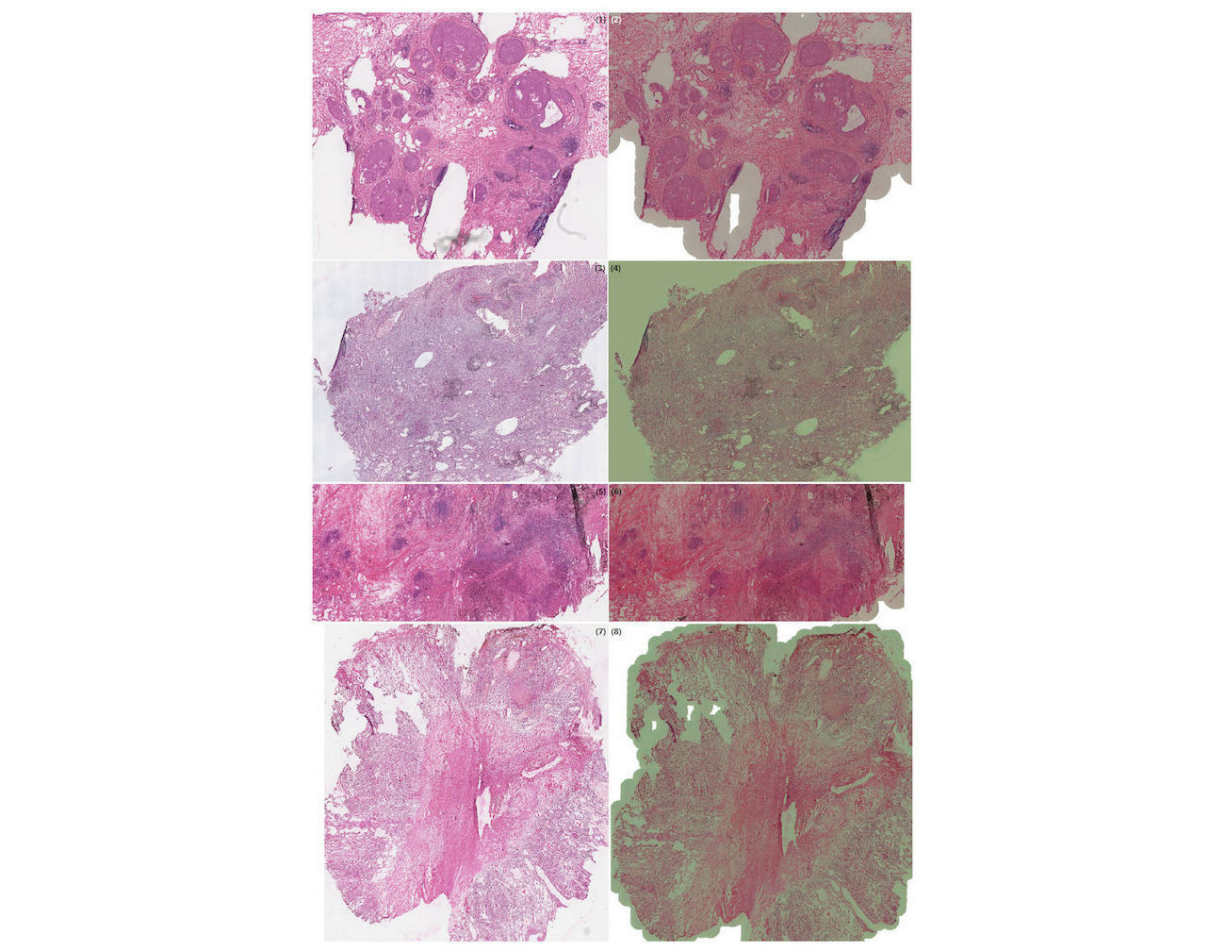
Selected virtual slide regions from sWSI (right) with poor quality, compared to those from the Leica scanner (left).

Using optical microscopy as the ground truth, the sWSI system achieved good performance similar to that of the standalone scanner in most sample categories except lung, as summarized in Table 4,Table 5,Table 6, and Table 7. For easier comparison, the per-category metrics are plotted in Figure 8, Figure 9, Figure 10, and Figure 11, with the average in Figure 12. Screen shots of VS regions where sWSI provided adequate or poor image quality are illustrated in Figure 13 and Figure 14. Although the performance was not ideal in absolute terms, all pathologists involved in this experiment firmly agreed that sWSI performed very well for these frozen section samples after viewing the results in retrospect. The pathologists also suggested that sWSI is clinically reliable for daily use on easier and more common samples, such as margins or paraffin sections, which are not covered in this experiment.

**Table 4:** Accuracy of diagnosis based on sWSI VS by pathologist A. Results are presented as ratios between 0.00 and 1.00.

Category	Accurate	LSen	LSpe
Breast	0.70 (14/20)	0.05 (1/20)	0.25 (5/20)
Uterine corpus	0.75 (15/20)	0.10 (2/20)	0.15 (3/20)
Thyroid	0.68 (19/28)	0.11 (3/28)	0.21 (6/28)
Lung	0.43 (13/31)	0.30 (9/31)	0.27 (8/31)
Ovary	0.00 (0/1)	1.00 (1/1)	0.00 (0/1)
Average	0.61 (61/100)	0.16 (16/100)	0.22 (22/100)

**Table 5:** Accuracy of diagnosis based on sWSI VS by pathologist B. Results are presented as ratios between 0.00 and 1.00.

Category	Accurate	LSen	LSpe
Breast	0.85 (17/20)	0.05 (1/20)	0.10 (2/20)
Uterine corpus	1.00 (20/20)	0.00 (0/20)	0.00 (0/20)
Thyroid	0.68 (19/28)	0.14 (4/28)	0.18 (5/28)
Lung	0.57 (17/31)	0.20 (6/31)	0.23 (7/31)
Ovary	0.00 (0/1)	0.00 (0/1)	1.00 (1/1)
Average	0.74 (73/100)	0.11 (11/100)	0.15 (15/100)

**Table 6:** Accuracy of diagnosis based on scanner VS by pathologist D. Results are presented as ratios between 0.00 and 1.00.

Category	Accurate	LSen	LSpe
Breast	0.95 (19/20)	0.05 (1/20)	0.00 (0/20)
Uterine corpus	0.95 (19/20)	0.00 (0/20)	0.05 (1/20)
Thyroid	0.85 (23/28)	0.00 (0/28)	0.15 (4/28)
Lung	0.77 (24/31)	0.16 (5/31)	0.06 (2/31)
Ovary	1.00 (1/1)	0.00 (0/1)	0.00 (0/1)
Average	0.87 (86/100)	0.06 (6/100)	0.07 (7/100)

**Table 7:** Accuracy of diagnosis based on scanner VS by pathologist E. Results are presented as ratios between 0.00 and 1.00.

Category	Accurate	LSen	LSpe
Breast	0.70 (14/20)	0.05 (1/20)	0.25 (5/20)
Uterine corpus	0.95 (19/20)	0.00 (0/20)	0.05 (1/20)
Thyroid	0.74 (20/28)	0.00 (0/28)	0.26 (7/28)
Lung	0.71 (22/31)	0.13 (4/31)	0.16 (5/31)
Ovary	0.00 (0/1)	0.00 (0/1)	1.00 (1/1)
Average	0.75 (75/100)	0.05 (5/100)	0.19 (19/100)

For validation purposes, all VSs in this experiment captured by sWSI and the scanner can be viewed online through the links provided in Multimedia Appendix 1. Details of each sample and the diagnosis, including the ground truth and those made based on standalone scanners or sWSI, are provided in Multimedia Appendix 2. Limited by time, this record is mostly in its original language (Chinese) but may be translated and requested from the authors.

### Learning Curve

**Figure 15:**
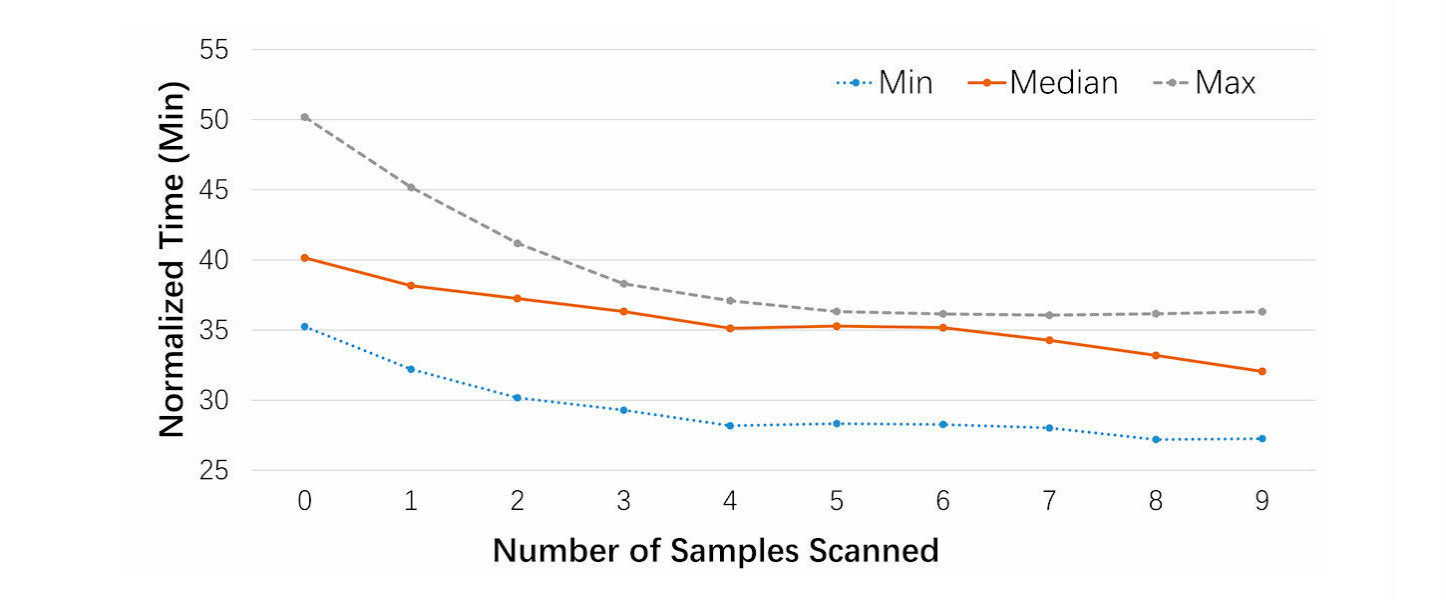
Normalized scan time versus number of samples scanned.

The learning curve, which is inversely approximated by the scan time normalized for a 15-by-15 mm sample being plotted against the number of samples scanned, is shown in Figure 15. In the experiment, productivity varied significantly among operators, as the slower ones consumed up to 50% more time at the beginning of the experiment. However, the slower operators caught up swiftly through practice and eventually reached just over 35 minutes for every 15-by-15 mm sample, which is comparable to automatic scanners. Most operators reached a stable level of proficiency after scanning just 5 slides. It should be noted that the 20X objective lens for frozen section samples is a relatively challenging application, as the samples are often uneven and require frequent focus adjustments. As a result, the scan speed in Figure 15 may be considered as a worst-case scenario.

## Discussion

### Current Limitations

First, a significant performance gap exists among different sample categories that are challenging and require very high image quality. Based on the experimental results, sWSI diagnoses of leiomyoma of the uterus, adenomyosis, papillary carcinoma of the thyroid, invasive breast carcinoma, and fibroadenoma of the breast are relatively more reliable than standard procedures, but those for follicular carcinoma of the thyroid, intraductal papillary neoplasms of the breast, and lung cancer are much worse. On the positive side, since the quality can be assessed after scanning and before inspection by the pathologists, these mistakes may be largely avoided in practice, especially those of LSpe (which should be analyzed by optical microscopy). However, this finding does reveal the fact that the current version of sWSI may not be suitable to all types of samples, or at least that testing may be required before formally adopting sWSI for applications requiring the highest quality images.

Second, similar to standalone scanners, sWSI is strongly affected by the physical preparation quality of the samples. Common problems include samples being broken into multiple pieces, varying thickness, and poor staining quality.

Finally, sWSI suffers from an inferior setup of hardware and environments, which comes with its focus on cost and scalability, and may make its competition against standalone scanners challenging. One such example is data storage: standalone scanners are mostly designed to work with high-speed wired and local networks. Since sWSI provides wireless connection-based data transmission and Web browser-based viewing, limits on bandwidth may severely affect image quality. Another example is that VSs produced by scanners are displayed on specialized work station monitors with high coverage of red/green/blue color space and 4K resolution, while those of sWSI are reviewed on normal monitors, or even smartphones with small screens.

The design of the experiment can be improved in future work. Based on discussions with participating pathologists, it can be safely assumed that there would be little error in diagnoses made by senior pathologists. However, from experimental results and comments, there are significant differences in the level of confidence that pathologists have regarding dubious cases where the provided VSs alone are not sufficient for a firm decision. In practical procedures, pathologists would undertake further investigation with other techniques, such as paraffin embedding and sectioning, which is more reliable (but far more time consuming) than frozen sectioning. In this experiment, participants were required to make binary decisions when judging thresholds that were different from each other. As a result, using multiple probabilities instead of a firm answer might be a better alternative.

### Future Work

There are a number of technical issues to be solved in future research and development. First, some parameters on Android phones cannot be controlled through publicly available programing interfaces for older Android operating systems. Weakened control may lead to improper configurations, such as a long exposure time causing blur. Second, the openGL driver which offers general-purpose graphic processing unit (GPGPU) computing potential is very difficult to work with and produces unexpected results on many smartphone models for unknown reasons. Preliminary research using GPGPU on iPhones brought a dramatic boost in processing speed over 60%, but certain models behave improperly. Finally, the real-time movement tracking is inaccurate and leads to location mismatch in subsequent mini-map construction. Although this issue does not affect the VS generation, the mis-drawing in the mini-map can be confusing.

Limited by time and development cost, the international version of sWSI is only offered on iOS. The Chinese version (Tai Rui Jing Xia), which has a complicated still-FoV capturing tool and clinical report system, supports both Android and iOS but may not be accessible from abroad. In follow-up studies, both versions will be evaluated and compared. More quantitative metrics may also be covered (eg, the measure of absolute image quality) even though most pathologist users suggest such measurements have very limited implications in clinical settings.

On the clinical side, only the basic scanning function of sWSI was evaluated in this experiment, leaving sWSI's many other useful functions to be tested. These functions include swift scanning at a lower magnification then adding static FoVs at higher magnification to cover both speed and detail, recording z-stacks of thick smears with video clips, and potential application of sWSI in fluorescent or dark-field microscopy. The validation of these functions, as well as experiments with expanded sample sizes, are planned in future work.

### Conclusions

In this paper, an ultra-low-cost WSI system named sWSI (with clients hosted on mainstream Android and iOS smartphones) was introduced and analyzed. Compared to automatic scanners and high-end computer-based solutions, this alternative dramatically reduces the setup cost to as low as US $100 per unit with service costs of US $1-$10 per scan. Although sWSI may not replace existing dedicated devices, it could become a reliable alternative that weighs more on cost-effectiveness.

By employing distributed image processing, both robustness and efficiency are covered. Through high performance computing and real-time feedback, user friendliness is optimized with minimal manual input, leaving most interface-kernel coordination (and even image distortion correction) fully automated. Based on clinical evaluation using 100 frozen section samples, sWSI is considered adequate by expert pathologists for making a diagnosis for most sample types. The overall accuracy based on sWSI VSs is slightly poorer than that based on high-end scanners, which is the current solution for digitizing whole slides. This gap is largely attributed to low specificity for samples requiring higher levels of detail (eg, lungs), which means that most of the inadequacy of sWSI comes from relatively low image quality, which can be identified before diagnoses are made.

In the experiment, 15 operators with no prior experience with sWSI learned to use the manual scanning very quickly. User throughput stably reached between 27 and 36 minutes per 15-by-15 mm sample area under a 20X objective lens after scanning 9 slides each, similar to that of mid-to-low-end scanners. In low-frequency usage situations (eg, remote or low-tier hospitals), this level of labor may be considered cost-effective, given the vast financial savings from deploying sWSI instead of scanners.

## Appendix

Multimedia Appendix 1 Links to virtual slides produced by sWSI and the Leica scanner.

Multimedia Appendix 2 Original diagnosis (Chinese).

## Abbreviations

FoV: field-of-view
GPGPU: general-purpose graphic processing unit
HOP: high-order polynomial
KP: key point
LSen: low sensitivity
LSpe: low specificity
SJTU-PC: Pathology Center, Shanghai General Hospital, School of Medicine, Shanghai Jiaotong
SURF: speeded-up robust features
sWSI: scalable Whole Slide Imaging
VS: virtual slide
WSI: whole slide imaging
